# Simultaneous Determination of Salicylic Acid, Jasmonic Acid, Methyl Salicylate, and Methyl Jasmonate from *Ulmus pumila* Leaves by GC-MS

**DOI:** 10.1155/2015/698630

**Published:** 2015-09-17

**Authors:** Zhi-hong Huang, Zhi-li Wang, Bao-lin Shi, Dong Wei, Jian-xin Chen, Su-li Wang, Bao-jia Gao

**Affiliations:** ^1^Agricultural University of Hebei, Baoding 071001, China; ^2^Hebei North University, Zhangjiakou 075000, China

## Abstract

Salicylic acid, jasmonic acid, methyl salicylate, and methyl jasmonate are important phytohormones and defensive signaling compounds, so it is of great importance to determine their levels rapidly and accurately. The study uses *Ulmus pumila* leaves infected by *Tetraneura akinire Sasaki* at different stages as materials; after extraction with 80% methanol and ethyl acetate and purification with primary secondary amine (PSA) and graphitized carbon blacks (GCB), the contents of signal compounds salicylic acid, jasmonic acid, methyl salicylate, and methyl jasmonate were determined by GC-MS. The results showed that the level of salicylic acid, jasmonic acid, methyl salicylate, and methyl jasmonate increased remarkably in *U. pumila* once infected by *T. akinire Sasaki*, but the maximums of these four compounds occurred at different times. Salicylic acid level reached the highest at the early stage, and jasmonic acid level went to the maximum in the middle stage; by contrast, change of content of methyl salicylate and methyl jasmonate was the quite opposite.

## 1. Introduction

When suffering herbivores attack in nature, in addition to relying on their stems and leaf hairs, spines, and other first physical barriers, plants also depend on the changes in their hormone levels or release of certain signal substances involved in chemically induced defense against attack [1]. Up to date, researches have shown that the plant hormones including salicylic acid, jasmonic acid, and their methylated products had a very important role in transmission of defense signal in plants [2]. Salicylic acid plays many physiological effects in plants, and many experiments have proved it is one of the key signal molecules produced by systemic acquired resistance. Jasmonic acid, widely found in higher plants, is a new type of plant hormone to make plants cause a variety of morphological or physiological effects. Its role is to induce the open of glumous flower and the formation of the tuber [3], to inhibit the pollen germination, root growth, and so on [4]. In addition, jasmonic acid can induce the plant to start the defense system by changes in endogenous levels when the plant is attacked by pathogens or herbivorous animals [5]. As endogenous signal molecules, their physiological effects are extremely complex in plants, and thus it is of great significance to establish a stable and sensitive method for determination of endogenous signal compounds.

The contents of salicylic acid and jasmonic acid are very low in plants, so the measurement is very difficult. With the development of separation and identification technology, our predecessors have applied a variety of methods for the determination of signal compounds in plants. Currently, the main methods of determination of salicylic acid and jasmonic acid are ELISA, HPLC, and UPLC [6]. With the method of ELISA, salicylic acid and jasmonic acid in samples get a better enrichment and then are analyzed by chromatography. In this way, the recovery rate of the sample is higher. However, it is difficult to produce monoclonal antibodies, so not easy to popularize. Besides, combined with triple quadrupole mass spectrometer [7–10], time-of-flight mass spectrometer, and fluorescence detector [11, 12], HPLC can measure jasmonic acid and salicylic acid levels simultaneously. Zhang et al. have determined jasmonic acid level from extract of* Hevea brasiliensis* bark by using capillary electrophoresis laser-induced fluorescence detection method [13]. But this method needs to treat jasmonic acid without fluorescence emission groups with fluorescence derivatization, making the analysis process more cumbersome. The advantage of GC-MS for compounds analysis is that it can be measured directly without derivation after a simple separation.

To the best of our knowledge, the measurement of signal compound levels of* Ulmus pumila* leaves has not been reported. In this paper, the method of extraction, purification, and GC-MS determination was established. Moreover, content of signal compounds in different periods after the attack of* Tetraneura akinire Sasaki* to* U. pumila* leaves was determined, and their changes were also analyzed. The aim of this study was to reveal change trends of endogenous jasmonic acid and salicylic acid levels in host* U. pumila* leaves during the formation of gall.

## 2. Experiment

### 2.1. Materials

Jasmonic acid, salicylic acid, methyl salicylate, methyl jasmonate, dihydrojasmonate (internal standard, IS), primary secondary amine, and graphitized carbon blacks were purchased from Germany sigma company, with the purities higher than 98.5%.

**Figure 1:**
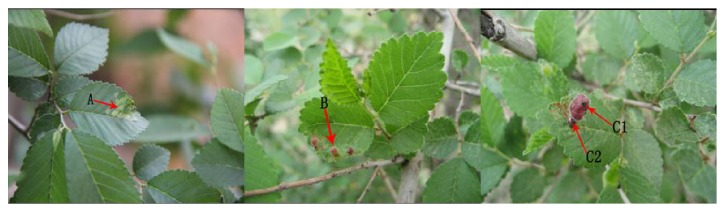
Three stages of* U. pumila* leaves infected by* T. akinire Sasaki*. A: initiation phase; B: growth phase; C: dehiscence phase.

Leaves of* U. pumila* at different infection stages including galls formation stage (Figure 1-A, early stage), growth (Figure 1-B, middle stage), and dehiscence (Figure 1-C1, C2, late stage) were collected from the countryside of Zhangjiakou City, Hebei, China. Meanwhile, healthy leaves were also collected as control samples in the same plants and same stage. All samples were placed in liquid nitrogen immediately after collection and then stored at −80°C refrigerator.

### 2.2. Preparation of Standard Solutions

Approximately 10 mg of individual standards, jasmonic acid, salicylic acid, methyl salicylate, and methyl jasmonate was dissolved in methanol to obtain standard stock solutions. The mix working solutions were prepared by diluting the appropriate volume of standard stock solutions with methanol. Both the stock solutions and working solutions were stored in dark at −20°C freezer.

### 2.3. Sample Preparation

All leaves in liquid nitrogen were placed in room temperature. After liquid nitrogen was evaporated, 1.0 g of samples was accurately weighed into a 50 mL into a centrifuge tube, and then 10 *μ*L internal standard of dihydro jasmonate (DHJA) and 10 mL of 80% cooled methanol (pH 2.5~3.0) were added. The mixture was homogenized using a blender for 2 min at 6000 rpm and then soaked overnight at 4°C. The next day, 10 mL ethyl acetate was added into the tube and vortexed for 1 min and then centrifuged for 10 min at 10,000/min at 4°C. The supernatant was collected and was added, 0.2 g GCB and 0.6 g PSA, and then vortexed for 1 min. After centrifugation at 5000 r/min for 5 min, the supernatants were collected and evaporated to dryness with a gentle stream of nitrogen and then dissolved in 1 mL ethyl acetate for further analysis.

### 2.4. GC-MS Conditions

The extract was injected into an Agilent 6890N-5973i GC/MS equipped with a DB-5MS (UI) chromatographic column (30 m × 0.25 mm × 0.25 *μ*m). The injection temperature and injected volume were set to 280°C and 2 *μ*L, respectively. The helium flow rate was 1.1 mL/min. The temperature program was as follows: the initial column temperature was set at 70°C for 4 min, increasing to 300°C at the speed of 10°C/min and lasting for 2 min and then increasing to 340°C at the speed of 5°C/min and holding until the end of analysis. Quantification was performed in the selected-ion monitoring (SIM) mode after electron ionization (70 eV) with a dwell time set at 0.3 s, and source temperature and quadrupole temperature were set at 230°C and 150°C, respectively. The mass data were collected in the range from* m/z* 40 to* m/z* 500.

### 2.5. Data Analysis

Experiment was conducted three times, and the experimental results were expressed in terms of the means ± standard deviation (SD). All experimental data were analyzed using SPSS 13.0. Analysis of variance was performed to compare the difference between the sample and the control.

## 3. Results and Discussion

### 3.1. Optimization of Sample Preparation

In order to optimize the sample preparation conditions, different extraction solvents (different ratios of MeOH or EtOH : H_2_O) and sorbents were investigated.  Table 1 showed the recoveries of all the compounds, which indicated that 80% methanol can get better extraction effect and their recoveries were higher than 70%. Therefore, 80% methanol was selected as the extraction solvent. In order to remove the interference of impurities from the extracts, the same volume of ethyl acetate and n-hexane were used as the extract solvent to evaluate the extraction efficiency of these signal compounds. The results showed that the ethyl acetate got better results (Table 1), which was chosen in further experiments.

Traditionally, PSA is commonly applied as an effective clean-up sorbent for polar organic acid removal, and GCB is used for pigment and sterols removal. To examine the impact of PSA and GCB on the purification effect of samples and the recovery of analytes, different amounts of PSA (0.4–0.8 g) mixed with GCB (0.1–0.3 mg) were used to purify the samples. As can be seen form Table 2, when 0.6 g of PSA and 0.2 g of GCB were used, the recovery rate was the highest. However, recovery values of salicylic acid and jasmonic acid decreased when GCB was above 0.2 g. It is probable that GCB adsorption mechanism with compounds whose structure has a high affinity toward GCB.

### 3.2. Optimization of Chromatography and Mass Spectrometry Conditions

**Figure 2:**
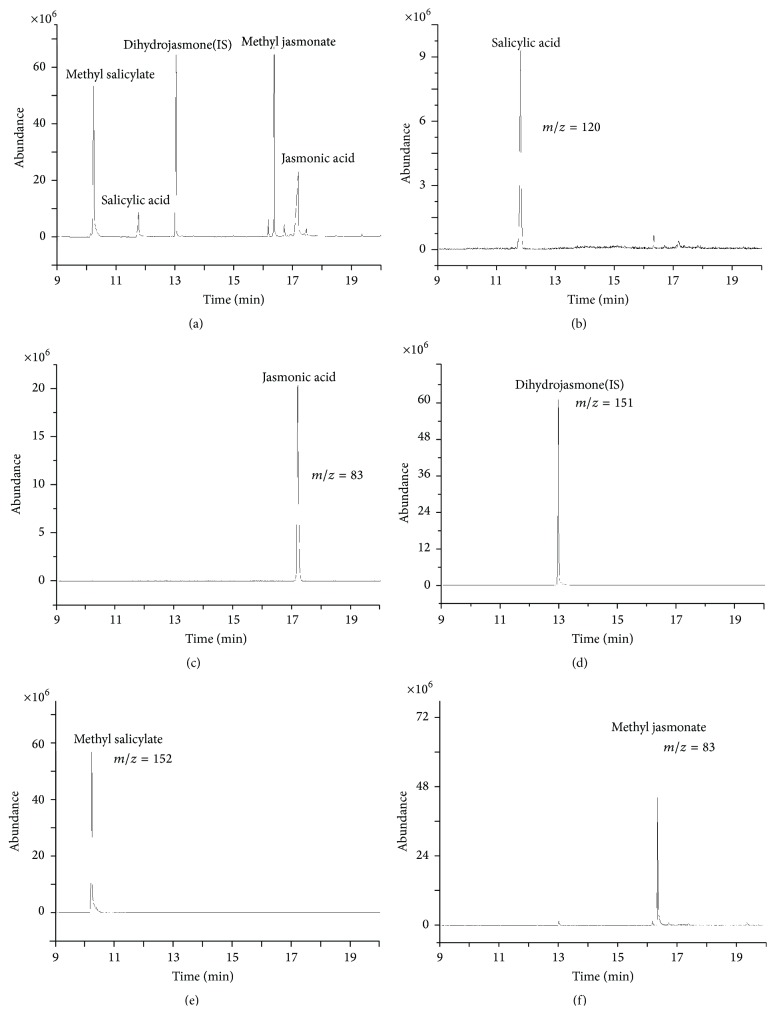
MS total ion (TIC) for 10 *μ*g/mg signal compounds and internal standard solution (a) and SIM chromatograms obtained from standard solution (b–f).

**Figure 3:**
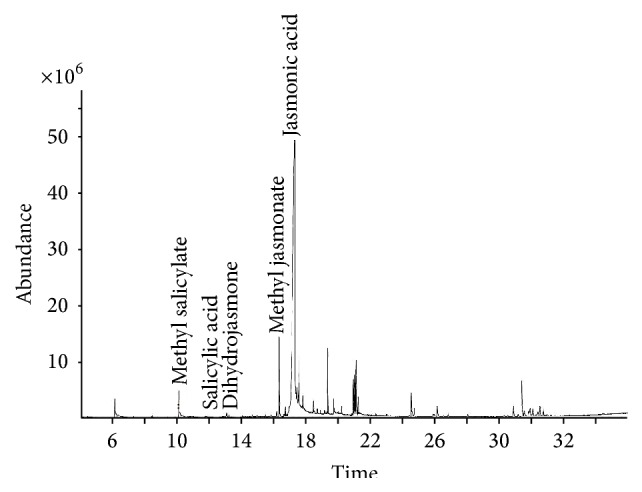
Typical chromatogram of defense chemical of* U. pumila* L. leaves after being infected by* Tetraneura akinire Sasaki*.

DB-5 (30.0 m × 320 *μ*m × 0.25 *μ*m) and HP-5 (30.0 m × 320 *μ*m × 1 *μ*m) capillary columns were tested to separate the target compounds. It was found that there were large differences on the separation efficiency in two capillary columns. Using HP-5 column, the peak of salicylic acid and methyl salicylate overlapped partly, and the separation degree of jasmonic acid and methyl jasmonate was not very good too, whereas, in DB-5 column, jasmonic acid and methyl jasmonate can be separated better and salicylic acid and methyl salicylate can also be completely separated. Based on these results, DB-5 column was used in further studies. Full scan was performed from 40 to 500 amu to determine the retention time and all major ions of each induced compound, and the results are given in Table 3. In the selected ion monitoring (SIM) mode, only one compound was scanned during each scan period to reduce the possibility of interference. MS total ion (TIC) for 10 *μ*g/mg signal compounds and internal standard solution (a) and SIM chromatograms obtained from standard solution were shown in Figure 2. The typical chromatogram of samples was shown in Figure 3.

### 3.3. Method Validation

#### 3.3.1. Calibration Curves and Limits of Detection and Quantification

Internal standard was used consistently for quantification in this method. A calibration curve of target compound was constructed by plotting analyte concentrations against ratios of IS (dihydrojasmonate) and analyte peak areas. The method validation for the quantification of 4 compounds showed good linearity and sensitivity. The squared correlation coefficients (*r* ^2^) were obtained for all of the four compounds ranging from 0.9976 to 0.9997. The limit of detection (LOD) and limit of quantification (LOQ) were calculated, respectively, with a signal-to-noise (S/N) of 3 and 10, respectively, and the results are seen in Table 4.

#### 3.3.2. Precision, Accuracy, and Repeatability

The precision of the method was validated by the determination of intra- and interday variance. The intraday precision was determined by replicate analysis (*n* = 6) of standard solutions of the four signal compounds at low, medium, and high concentrations in a single day, while the interday values were obtained over three consecutive days. The concentration of each solution was determined using a calibration curve prepared on the same day. The intraday precision and interday precision calculated as RSD were within the range of 1.14% to 4.42% and 0.37% to 4.02%. The results were presented in Table 5.

Recovery was used to further evaluate the accuracy of the method. Known amounts of each standard solution at three different concentration levels were mixed with known amounts of* U. pumila* samples; the samples were then extracted and analyzed with the above established method. The experiments were repeated three times at each level. The results showed that recovery ranged from 73.8% to 103.0%, and RSD ranged from 2.14% to 11.05%. Details have been listed in Table 6.

Six* U. pumila* samples from the same source were extracted and analyzed using the above established method. The RSD values were calculated as a measurement of method repeatability. RSD values of salicylic acid, jasmonic acid, methyl salicylate, and methyl jasmonate were 1.22%, 1.98%, 1.67%, and 2.70%, respectively, which showed high repeatability.

### 3.4. Sample Analysis

Insect gall is that when aphids (*Tetraneura akinire Sasaki*) feed on juice in the phloem of* U. pumila* leaves, and leaves suffer the stimulation of its secretions, and then the cells divide rapidly and differentiate abnormally, leading to cell proliferation around vascular and formation of abnormal nodules or protrusions [14]. Studies have shown that the development of galls can be divided into three periods, which are the initiation phase (early stage), growth phase (middle stage), and dehiscence phase (late stage). A series of physiological and biochemical reactions in the plant is produced in the process of galls formation, leading to changes in the levels of nutrient and signal compounds and variations in the activity of defensive enzymes.

**Figure 4:**
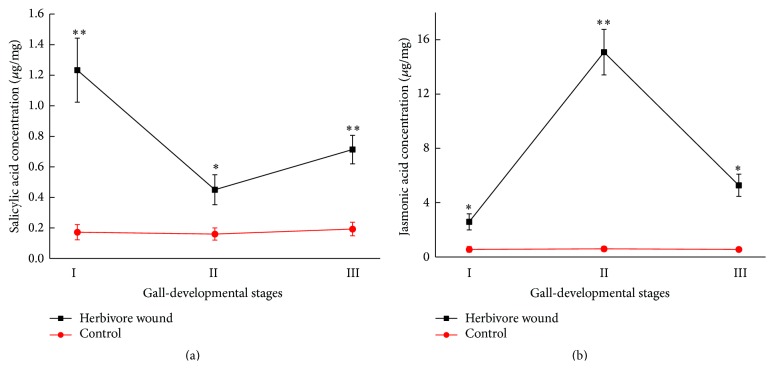
Changes in concentrations of salicylic acid (a) and jasmonic acid (b) in* U. pumila *leaves infected by* Tetraneura akinire Sasaki*. *∗* and *∗∗* represent significant difference compared with control at *P* < 0.05 and *P* < 0.01 level, respectively.

Changes in the levels of salicylic acid and jasmonic acid in* U. pumila* leaves infected by* Tetraneura akinire Sasaki *were determined using methods as described above. As can be seen in Figure 4, the changes of salicylic acid level during insect wounding were striking (*P* < 0.05). At early stage of infection, salicylic acid level was remarkably higher (*P* < 0.01), and the peak value was up to 6.2-fold higher compared to that of control, and then it was followed by a sharp decline, while the level of jasmonic acid at early stage was slightly higher than that in control. With the infection going on, jasmonic acid was significantly higher in the middle stage (*P* < 0.01), then reached its peak (up to 24-fold higher) compared to the control, and finally decreased.

**Figure 5:**
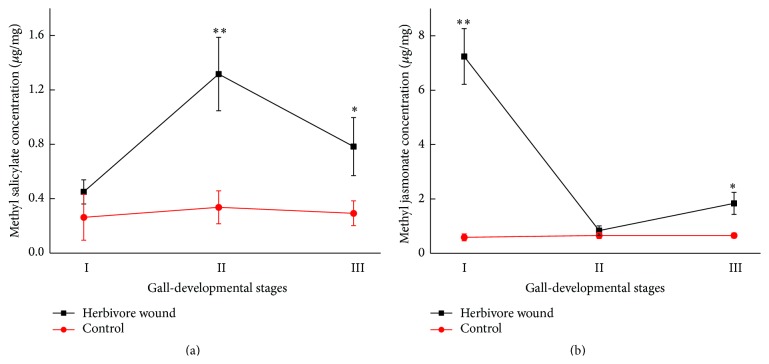
Changes in concentrations of methyl salicylate (a) and methyl jasmonate (b) in* U. pumila* leaves infected by* Tetraneura akinire Sasaki*. *∗* and *∗∗* represent significant difference compared with control at *P* < 0.05 and *P* < 0.01 level, respectively.

Changes in the levels of methyl salicylate and methyl jasmonate in* U. pumila* leaves infected by* Tetraneura akinire Sasaki *are given in Figure 5. Their levels varied greatly in different periods of infection (*P* < 0.05). At early stage, methyl salicylate level was low and close to the control, then gradually increased to the highest (up to 2.9-fold higher than control group) in the middle stage (*P* < 0.01), and declined sharply soon afterwards. Whereas methyl jasmonate level was significantly higher than one in the control at early stage (*P* < 0.01), as the time going on, it decreased gradually to the minimum value approaching the control in the middle stage and then increased sharply.

## 4. Conclusion

In the present study, optimized extraction and purification methods for extraction of endogenous signal compounds from* U. pumila* leaves have been developed. And a simple, rapid, and sensitive GC-MS method was established for the quantification of four signal compounds. The proposed method can be used for the determination of these endogenous compounds in* U. pumila* leaves.

## Figures and Tables

**Table 1:** Effect of extract solvent on extraction efficiency (yield %).

Compound	MeOH/H_2_O			EtOH/H_2_O			MeOH/H_2_O (80 : 20) + ethyl acetate	MeOH/H_2_O (80 : 20) + n-hexane
	90 : 10	80 : 20	70 : 30	90 : 10	80 : 20	70 : 30		
Salicylic acid	69.5	84.4	75.4	58.3	83.5	72.6	80.4	68.5
Jasmonic acid	67.8	88.5	72.6	56.6	79.8	82.4	85.3	70.8
Methyl salicylate	79.9	99.8	83.6	82.5	95.6	90.5	94.6	85.6
Methyl jasmonate	84.5	94.5	85.6	93.4	99.7	93.5	95.3	83.5

**Table 2:** Recoveries using different amounts of PSA and GCB (%).

Compound	0.4 g PSA + 0.1 g GCB	0.6 g PSA + 0.1 g GCB	0.6 g PSA + 0.2 g GCB	0.8 g PSA + 0.2 g GCB	0.6 g PSA + 0.3 g GCB	0.8 g PSA + 0.3 g GCB
Salicylic acid	82.2	84.6	86.1	83.9	69.7	73.2
Jasmonic acid	85.7	83.8	88.4	81.7	72.5	68.5
Methyl salicylate	97.8	94.5	98.8	94.2	88.4	85.5
Methyl jasmonate	99.2	98.6	99.7	98.6	85.8	87.9

**Table 3:** MS parameters for analysis of four compounds.

Compounds	Retention time (min)	Quantification (*m*/*z*)	Identification (*m*/*z*)
Salicylic acid	11.84	120	94,138
Jasmonic acid	17.12	83	151,210
Dihydro jasmonate	13.01	151	194,164
Methyl salicylate	10.23	152	120,92
Methyl jasmonate	16.35	83	110,67

**Table 4:** Linear regression data and validation for four compounds.

Compounds	Linear ranges (*μ*g·mL^−1^)	Calibration equation	*r* ^2^	LOD (*μ*g·mL^−1^)	LOQ (*μ*g·mL^−1^)
Salicylic acid	0.12–12.0	*y* = 1.0309*x* − 0.0251	0.9997	0.24	0.78
Jasmonic acid	0.11–11.0	*y* = 17.060*x* − 0.3347	0.9986	0.12	0.38
Methyl salicylate	0.14–14.0	*y* = 1.0291*x* + 0.0206	0.9976	0.05	0.20
Methyl jasmonate	0.12–12.0	*y* = 1.0135*x* + 0.0004	0.9996	0.03	0.12

**Table 5:** Intra- and interday precision for the four compounds (*n* = 6).

Compounds	Concentration	Intraday precision	Interday precision
	(*μ*g/mL)	(RSD, %)	(RSD, %)
Salicylic acid	0.24	4.42	4.02
	1.20	3.83	2.47
	6.00	2.25	2.15

Jasmonic acid	0.22	2.47	1.75
	1.10	2.71	1.44
	5.50	1.96	1.33

Methyl salicylate	0.14	1.63	2.26
	0.70	1.88	1.64
	3.50	1.14	0.37

Methyl jasmonate	0.12	1.28	2.35
	0.60	1.79	1.17
	3.00	1.94	1.68

**Table 6:** Recovery of four signal compounds from spiked samples at three concentrations.

Compounds	Added (*μ*g·mg^−1^)	Measured (*μ*g·mg^−1^)					Recovery (%)	Precision (RSD, %)
Salicylic acid	1.0	0.87	0.77	0.84	0.78	0.65	78.2	10.39
	5.0	4.85	4.32	4.08	4.10	4.89	89.0	9.15
	10.0	9.87	8.94	8.80	9.52	8.97	92.2	4.91

Jasmonic acid	1.0	0.88	0.74	0.68	0.69	0.70	73.8	11.05
	5.0	4.02	4.28	4.52	4.87	4.68	89.5	7.54
	10.0	9.89	8.97	9.21	9.35	8.62	92.1	5.04

Methyl salicylate	1.0	0.94	0.91	0.88	0.91	0.85	89.8	3.76
	5.0	4.91	4.57	4.87	4.98	4.08	93.6	7.68
	10.0	10.25	10.67	9.75	11.21	9.63	103.0	6.26

Methyl jasmonate	1.0	0.95	0.91	0.89	0.98	0.98	94.21	4.38
	5.0	4.85	4.96	4.89	4.91	5.12	98.92	2.14
	10.0	10.69	9.97	9.85	9.05	9.27	97.66	6.52
